# Supplementary material for: Hospital segregation, critical care strain, and inpatient mortality during the COVID-19 pandemic in New York City
Source: PLoS One. 2024 Apr 11;19(4):e0301481. doi: 10.1371/journal.pone.0301481 (PMC11008816; doi:10.1371/journal.pone.0301481)
Supplement: Attachment — Submitted filename: Response to Reviewers.docx [file pone.0301481.s002.docx]

**Response to Reviewers**

Academic Editor Review:
Thank you for submitting this interesting manuscript to PLOS One. I would like to raise the following critiques for your consideration.

This in an interesting manuscript that examines whether hospitals in NYC that serve a higher proportion of Medicaid and uninsured patients experienced greater strain and hospital mortality during the 1st wave of the COVID pandemic. Not surprisingly, the authors found that greater ICU strain was associated with higher mortality. They also present data to show that hospitals that serve more under-served patients (divided by quartiles) had higher mortality rates. Although the findings are interesting, there are several critiques which should be addressed:

Major issues:
1) Based on the description of the statistical analysis plan, it is not clear whether this is a descriptive analysis or whether there was an a priori hypothesis about the mortality across hospital quartiles. This needs to be specified. If there was a pre-specified hypothesis, the appropriate use of statistical tests that examine trends across the quartiles should be used and described.

Thank you for raising this issue. We have now stated the hypotheses for the study at the end of the Introduction, which reads (page 5, lines 105-110): “Due to care segregation and a greater likelihood of COVID-19 infection and hospitalization among Medicaid-insured and uninsured individuals, we hypothesized that (1) hospitals serving a higher proportion of Medicaid and uninsured patients pre-COVID experienced a greater surge of patients requiring critical care and higher mortality rates during the first COVID-19 wave, and (2) the greater surge in critical care patients was positively associated with patient mortality.”

The statistical tests for these hypotheses are specified in the Methods section (pages 7-9).

2) For mortality (both ICU and non-ICU), the authors present relative changes compared to baseline (prior to pandemic). While this provides some interesting information, one can argue that the best outcome to see if there were truly differences in mortality across the hospital quartiles would be risk-adjusted mortality rates (not changes from baseline). These should also be reported in absolute values (i.e. mortality rate) rather than changes from baseline. COVID pneumonia was novel, so it is not clear that changes from a baseline (in which there was no COVID pneumonia) is as relevant as absolute mortality. When one looks at mortality trends during Wave 1 (Supplemental Table 2, Overall in-hospital deaths) across the hospital quartiles, it is not clear that there is an important trend or difference. The authors need to provide some clarity on these trends.

Thank you for this very helpful comment. Instead of comparing changes in mortality rates between the first COVID wave and baseline across hospital quartiles, we have followed your suggestion by focusing on the comparisons of crude and risk-adjusted mortality rates during the first COVID wave. The results for these comparisons are shown in Table 3. As presented in Table 3, the risk-adjusted mortality rates for ICU admissions were 36.4 (CI=34.7,38.2), 43.6 (CI=41.5,45.8), 45.9 (CI=43.8,48.1), and 45.7 (CI=43.6,48.0) per 100 admissions, and the risk-adjusted mortality rates for non-ICU admissions were 8.6 (CI=8.3,9.0), 10.9 (CI=10.6,11.3), 12.6 (CI=12.1,13.0), and 12.1 (CI=11.6,12.7) per 100 admissions by hospital quartile 1-4, respectively. T-tests showed that Q2-Q4 hospitals have statistically higher risk-adjusted ICU mortality rates than Q1 hospitals, and Q3-Q4 hospitals have statistically higher risk-adjusted non-ICU mortality rates than Q1 and Q2 hospitals. These findings are consistent with the hypothesis that hospitals serving a larger proportion of Medicaid and uninsured patients pre-COVID experienced higher mortality rates during the first COVID-19 wave, possibly due to hospital-level factors such as strained capacity.

3) The figures are very difficult to read (resolution) so it was challenging to understand the results. The supplementary tables are helpful, but there is an overwhelming amount of data. The authors should improve the clarity and quality of the figures (more informative figure legends would also be helpful) or provide tables that show key results in a way that readers could easily interpret.

Thank you for pointing out these issues. We agree that data presentation in the original submission was not as clear as it could have been. As an attempt to improve data clarity, we have followed your suggestion and replaced Figures 1, 2, and 5 in the original submission with Tables 1-4 that are hopefully easier to interpret. We would still like to keep Figures 3 and 4 from the original submission in the manuscript.

The resolution of the Figures seemed fine on our end, but somehow became blurry when they were combined with the text file during the online submission process. We communicated this issue with a Customer Care Coordinator from PLOS ONE and below is her response:

“Unfortunately, the reduction in resolution is a part of the PDF building process that cannot be prevented, and the PDF version of manuscripts will always contain a compressed version of very high-resolution figures. However, the files are available to download in their original resolution in the manuscript’s File Inventory. 

Since reviewers and the Academic Editor will have access to your File Inventory, they can download the files in their original resolution as necessary while evaluating the manuscript. If the manuscript is accepted, the figures or images in question will be linked in their full resolution.” 

Minor Issues:
1) Please provide a brief justification for why Wave 1 was used and not the entire duration of the COVID pandemic (or include additional waves)

The justification for the focus on Wave 1 has now been provided (Page 8, lines 169-171): “We focused on the first COVID-19 wave and a preceding period of similar length since reports and studies suggest that hospital capacity in NYC was particularly strained in the first wave of the pandemic due to a surge of critically ill patients.^16-18^”

2) In the statistical analysis section (page 7, lines 152-153), the authors state that “Possible changes in the composition of admissions between the two periods were also examined.” Please clarify what was examined and how.

We have replaced this sentence with the following (Page 8, lines 185-187): “We also provided information on the characteristics of admissions and deaths from the baseline and Wave 1 for all hospital quartiles to better understand distributions of COVID-19 related burdens among various demographic groups.”

3) Please provide some more details on how patient acuity and severity of illness are captured in the database (Page 7, lines 161-162)

We included two measures related to patient acuity: (1) whether an admission came through emergency department, and (2) severity of illness (SOI). More details on SOI have been provided in the text as follows (Page 9, lines 195-197): “SOI present on admission is based on All Patient Refined Diagnosis Related Groups (APR-DRG) classification and coded as 1=minor, 2=moderate, 3=major, and 4=extreme.”

4) It might be more appropriate to discuss how this work fits into the existing body of literature rather than offering recommendations to reduce care segregation (Discussion, Page 10-11). The paragraph on how this data might inform preparedness and response in future pandemics is excellent.

Thank you for the suggestion. We added a paragraph on how this analysis complements the existing literature (Page 13, lines 298-311). We would still like to keep the paragraph on care segregation since care segregation is a focus of the study and likely a contributor to the disparities in outcomes as found by the analysis.

Review Comments to the Author

Please use the space provided to explain your answers to the questions above. You may also include additional comments for the author, including concerns about dual publication, research ethics, or publication ethics. (Please upload your review as an attachment if it exceeds 20,000 characters)

Reviewer #1: The current study describes an important and robust analysis of hospitalization and mortality trends in NYC hospitals during the height of the COVID-pandemic. This analysis is innovative and clearly portrays disparities in hospital burden and negative outcomes associated with safety net hospitals that serve the majority of poor and un or under insured patients. I commend the authors on this thoughtful and important study. I only have some minor suggestions for some clarifications as follows:

1. It may be helpful to explicitly introduce the H+H system in NYC, which is the primary public and safety-net hospital in NYC. This may not be typical of other cities or well-known to non NYC readers, even though many of the highest risk hospitals in the study were part of the system.

Thank you for the suggestion. We have now explicitly introduced H+H as follows (Page 10: 218-219): “Almost all public hospitals in NYC are part of the H+H system, which is run by the city and a primary provider of care for Medicaid and uninsured patients.”

2. It is not immediately clear how to interpret patients with "self-pay" - in these data, would this be indicative of being uninsured or under-insured? Technically some very wealthy patients may "self-pay" as well, so would be helpful to explain to the reader how to interpret this and why authors decided to group this type of payment with Medicaid when creating the quartiles.

Thank you for raising this excellent point. While some self-pay patients may be well off and could access care provided at any hospital, the data showed that self-pay patients as a group perhaps have the most limited access to well-resourced facilities, as only 35% received inpatient care at AMCs and affiliates, the smallest proportion among all primary payer types. Based on 2017-2019 SPARCS, the table below (not included in the manuscript) illustrates this:

| **Hospital Type** | **Medicaid** | **Medicare** | **Private** | **Self pay** | **Other** | **Total** |
| --- | --- | --- | --- | --- | --- | --- |
| Private_AMC | 21% | 31% | 45% | 16% | 21% | 30% |
| Private_AMC_affiliated | 26% | 35% | 35% | 19% | 41% | 32% |
| Private_other | 26% | 21% | 12% | 18% | 14% | 20% |
| Public | 28% | 13% | 9% | 47% | 24% | 18% |
| Grand Total | 100% | 100% | 100% | 100% | 100% | 100% |

We have added the following in the text to explain the inclusion of self-pay admissions as a focus in the study (Page 6, lines 134-137): “Along with Medicaid insured admissions, we focused on self-pay admissions since self-pay patients as a group had the highest concentration of care received from public and private independent hospitals (> 65% in 2017-2019 SPARCS), suggesting limited access to well-resourced facilities among this population.”

3. Authors note in the methods "Several alternative proxies for hospital strain were used to assess sensitivity of regression estimates to hospital strain measures" while the results are included in supplementary tables, it would be helpful if authors explicitly listed what was tested.

Thank you for suggesting this. We have now provided details about the alternative proxies for hospital strain toward the end of the Methods section (Page 9, lines 202-207).

4. In the results, it would be helpful to start with the total number of hospitals that were examined and how many were distributed across quartiles, to get a sense of spread and volume of hospitals.

Thank you for the suggestion. We have added the following sentences in the beginning of the Results section to discuss the total number of hospitals and the proportion of all NYC hospitalizations provided by hospitals in each quartile (Pages 9-10: lines 213-218): “After excluding specialty hospitals and hospitals without provision of intensive care, a total of 47 hospitals in three categories – private AMC and affiliates, private other, and public – were included for the study. Q1 hospitals, almost all of which were AMCs or AMC affiliates, provided the largest proportion of all admissions in NYC at 33% (802,657/2,437,334). The proportion of admissions provided by each quartile steadily declined to 19% (464,936/2,437,334) among Q4 hospitals, all of which were public or private independent.”

5. Authors say "patients of color" when describing some of the results, but they never indicate what race/ethnicity groups they are included in this definition.

Thank you for pointing this out. We have replaced “patients of color” with “Hispanic and NH Black patients” throughout the Results section.

6. On line 206 authors say most regression estimates were consistent with previous studies - what previous studies is this referring to? Please reference appropriately

Since previous studies have conflicting results for certain estimates, such as those for race/ethnicity, we decided to present each statistically significant estimate (Pages 11-12, lines 265-274), instead of simply stating that these estimates were consistent with previous studies. We did not discuss variables not related to critical care strain in more depth since they were not the focus of the analysis.

7. All the figures are blurry and I am not able to see or read the labels - please produce higher quality images.

Our apologies for the blurry Figures. As discussed with the Editor above, the resolution of the Figures seemed fine on our end, but somehow became blurry when they were combined with the text file during the online submission process. We have communicated this issue with a Customer Care Coordinator from PLOS ONE and below is her response:

“Unfortunately, the reduction in resolution is a part of the PDF building process that cannot be prevented, and the PDF version of manuscripts will always contain a compressed version of very high-resolution figures. However, the files are available to download in their original resolution in the manuscript’s File Inventory. 

Since reviewers and the Academic Editor will have access to your File Inventory, they can download the files in their original resolution as necessary while evaluating the manuscript. If the manuscript is accepted, the figures or images in question will be linked in their full resolution.” 

8. The figures and tables hold an overwhelming amount of information - I may suggest presenting findings only by non-ICU and ICU and remove the third "all hospitalization" categories, to reduce the amount of information being presented and interpreted.

We agree that an overwhelming amount of information was presented in a way that’s not as clear as possible. The Editor also pointed out this issue above. We have replaced Figures 1, 2, and 5 from the original submission with Tables 1-4 to simplify data presentation. Hopefully, the data are now easier to interpret.
